# Partial Amelogenesis Imperfecta: A Report of a Rare Case

**DOI:** 10.7759/cureus.73856

**Published:** 2024-11-17

**Authors:** Hamad N AlBagieh, Lama M Alomran, Fatima Y AlBishry, Turki Khalid Aloraini, Nasser M Almofarej, Nawaf H Alhamzah, Meshal M Alqahtani

**Affiliations:** 1 College of Dentistry, King Saud University, Riyadh, SAU; 2 Department of Dentistry, King Saud University Medical City, Riyadh, SAU

**Keywords:** amelogenesis imperfecta, case report, enamel disorder, rare condition, saudi arabia

## Abstract

Amelogenesis imperfecta is a collection of genetic disorders that impair the structure of dental enamel. The condition presents in a variety of ways, affecting enamel development, mineralization, and maturation. Amelogenesis imperfecta can follow various inheritance patterns, including autosomal dominant, autosomal recessive, sex-linked, and sporadic. This case report describes a 22-year-old female diagnosed with amelogenesis imperfecta. The patient's clinical presentation, diagnostic process, and comprehensive treatment approaches are discussed, emphasizing the importance of multidisciplinary care in improving functional and esthetic outcomes. Treatment is ongoing and aims to improve both functional and esthetic outcomes.

This case report details the presentation, characteristic radiographic findings, and management discussion of a patient presented with a rare condition that is termed "partial amelogenesis imperfecta."

## Introduction

Amelogenesis imperfecta is the term for a group of genetically based disorders that impact the structure and clinical appearance of almost all teeth's enamel in a similar way [1]. This condition leads to defects in the formation of enamel, resulting in a significant reduction in its structural integrity and compositional quality. It also manifests as dental enamel that is markedly thin, hypersensitive, and esthetically unappealing. Furthermore, amelogenesis imperfecta can affect both deciduous and permanent dentition [2].

Amelogenesis imperfecta exhibits a diverse range of inheritance patterns, including autosomal dominant, autosomal recessive, sex-linked, and sporadic modes of transmission, as well as sporadic cases [3]. Also, amelogenesis imperfecta is characterized clinically by a spectrum of dental anomalies, including a yellow to brown discoloration, heightened susceptibility to dental caries and calculus deposition, accelerated attrition, gingival hyperplasia, and occasionally an anterior open bite. These manifestations are attributed to gene mutations in ENAM, AMEL, DLX3, and P63, which play crucial roles in tooth development and enamel formation [4].

Amelogenesis imperfecta is a relatively uncommon condition in Saudi Arabia, with a reported prevalence of approximately 1 in 333 individuals [5]. Moreover, amelogenesis imperfecta type I hypoplastic is considered the most common phenotype [6], which makes up 60-73% of amelogenesis imperfecta cases [7].

This study aims to evaluate the clinical characteristics, diagnostic process, and comprehensive treatment approaches for a patient with a rare case of partial amelogenesis imperfecta, emphasizing the importance of multidisciplinary care in enhancing functional and esthetic outcomes.

## Case presentation

A 22-year-old female presented to the Primary Care Unit at King Saud University Dental Hospital Clinics in Riyadh, Saudi Arabia, and was subsequently referred to the Department of Family Dentistry for comprehensive evaluation and treatment. The patient attended with a chief complaint of yellowish-brown discoloration with destructive teeth and sensitivity asking for a long-term esthetic solution, the patient's medical history disclosed no abnormalities nor medications or any allergies. The patient affirmed that there was no history of similar dental conditions within the family. Her past dental history exhibit that she did not experience this condition in her primary teeth. Moreover, she had undergone restorative procedures for dental caries. Oral hygiene is seriously impaired due to the difficulties associated with cleaning the multiple plaque-retentive areas. A written consent was obtained from the patient upon clinical photography and publication agreement.

**Figure 1:**
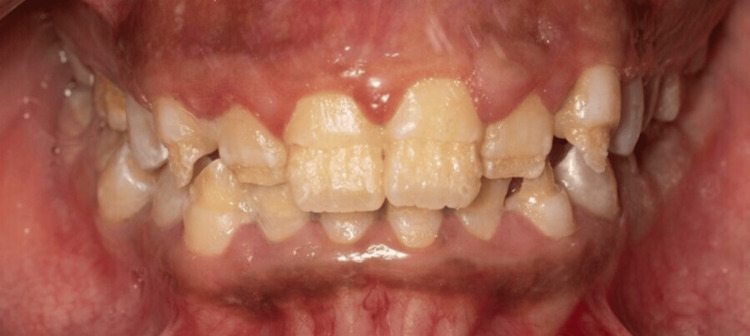
Intraoral frontal photo. Upper and lower anterior teeth showing thin, rough, and flaked enamel extended to the middle third with intact cervical third and exposed dentin.

**Figure 2:**
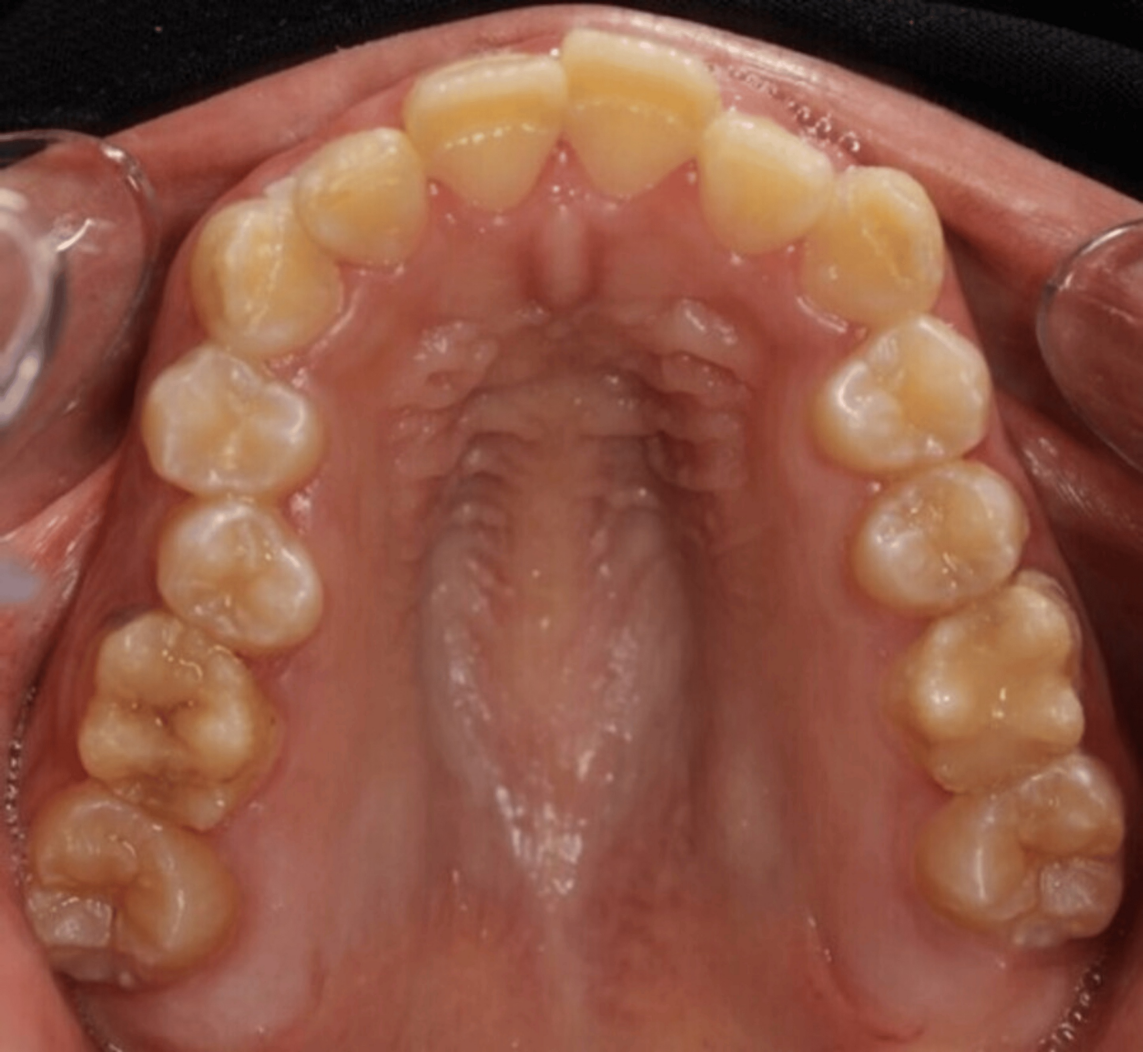
Intraoral occlusal photo of the upper jaw. Upper occlusal view showing affected palatal surface of upper anterior teeth.

**Figure 3:**
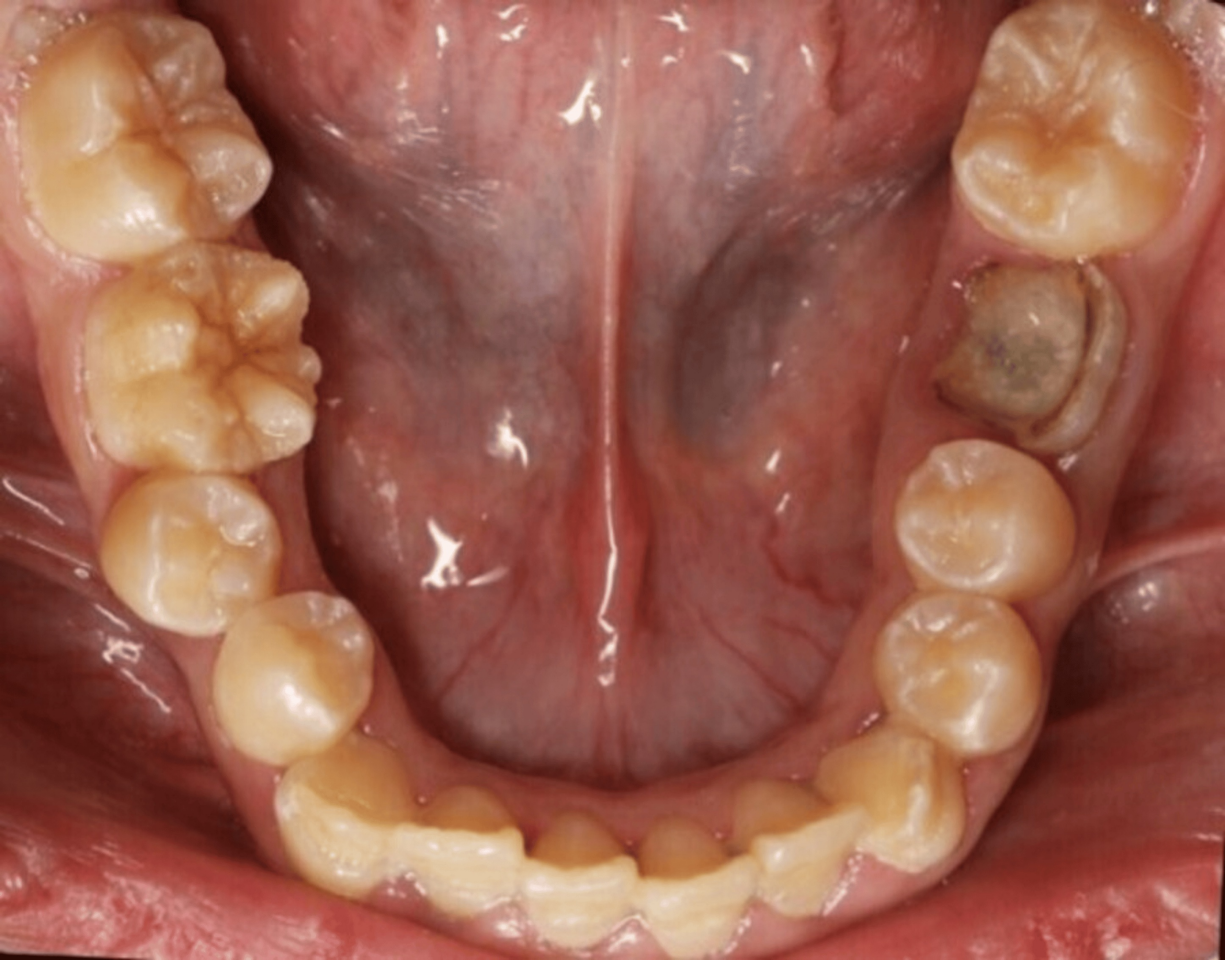
Intraoral occlusal photo of the lower jaw. Lower occlusal view showing affected labial surface of lower anterior teeth and badly broken lower left first molar.

**Figure 4:**
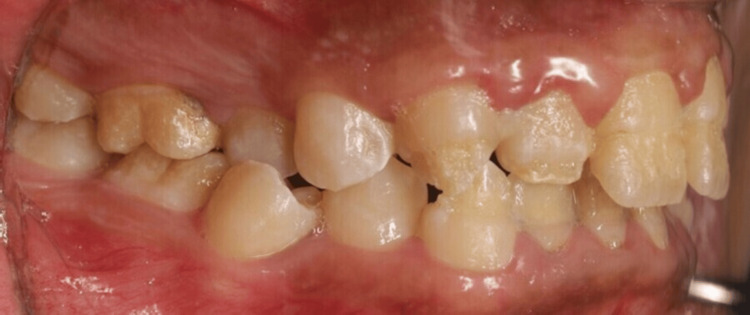
Intraoral lateral photo of the right side. Right lateral view showing affected labial surface of upper and lower teeth extended to the middle third with intact cervical third and exposed dentin.

**Figure 5:**
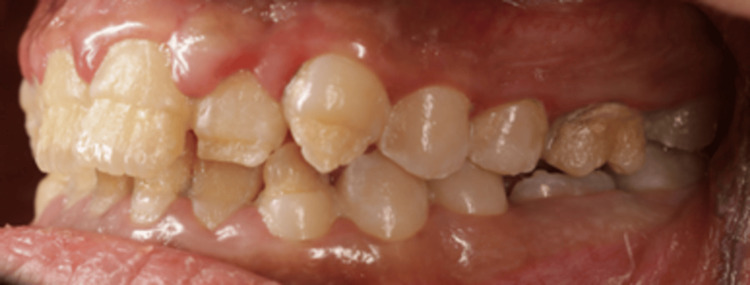
Intraoral lateral photo of the left side. Left lateral view showing affected labial surface of upper and lower teeth extended to the middle third with intact cervical third and exposed dentin.

**Figure 6:**
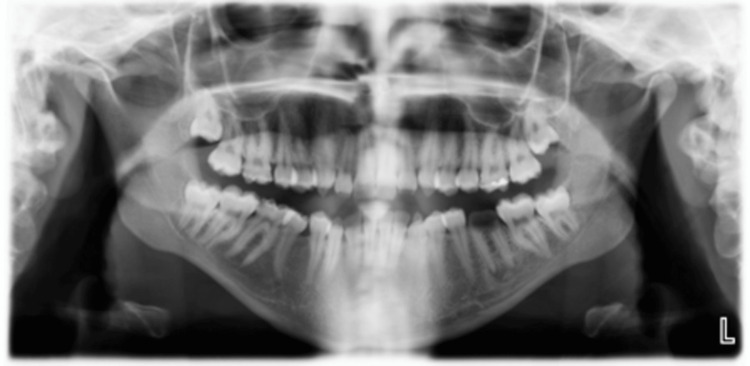
Panoramic radiograph. The panoramic radiograph reveals irregularities and a reduction in enamel density.

The extra-oral examination revealed a clicking sound in the temporomandibular joint; all other findings were within normal limits. Intra-oral examination indicated an increased overbite and overjet. Teeth demonstrated thin, rough, and flaked enamel extended to the middle third with exposed dentin affecting all teeth as shown in Figures 1-5. Multiple carious lesions were detected in the posterior teeth, and the periodontal evaluation revealed generalized plaque-induced gingivitis associated with gingival overgrowth. The diagnosis of generalized plaque-induced gingivitis is more the result of unsatisfactory personal oral hygiene due to the difficulty in cleaning all these imperfections on the dental crowns. The gum enlargement should be a consequence of the chronic inflammatory process of the gingiva. The panoramic radiograph shown in Figure 6 reveals irregularities and a reduction in enamel density.

The patient was referred to the Department of Oral Medicine to exclude any developmental disorders or syndromes and to the Department of Orthodontics for further assessment of the case.

Depending on the obtained data, the following dental treatment plan phases were presented and discussed with the patient: the disease control phase involved a multidisciplinary medical consultation to assess overall health and identify any contributing factors to oral disease. Scaling and prophylaxis to remove plaque and calculus, preventing further periodontal disease. Oral hygiene instructions to educate the patient on proper brushing and flossing techniques. Caries removal and restoration using resin-modified composite to restore tooth function and esthetics. Tooth #36 prognosis is hopeless and extraction with socket preservation is to be prepared for future implant placement.

The definitive treatment phase will be focused on implant placement to replace the missing tooth and provide a stable foundation for future restorations. Full-mouth rehabilitation is planned using indirect porcelain crowns, restoring the patient's teeth to their optimal function, esthetics, and health.

## Discussion

Diagnosis of amelogenesis imperfecta entails a comprehensive evaluation that involves establishing a probable inheritance pattern, identifying the specific phenotypic characteristics associated with amelogenesis imperfecta, and correlating these features with the developmental timeline of tooth formation to exclude any chronological developmental disturbances [3].

Amelogenesis imperfecta usually follows a genetic pattern; hence, some of the family members who preceded might have already established the diagnosis [8]. The patient may be unaware of any previous family medical history. Under these circumstances, the diagnosis and course of treatment are informed only by clinical and radiographic features, although genotype-based techniques should also be employed when possible [9].

Based on the enamel pattern and the stage of teeth involvement, amelogenesis imperfecta is divided into three categories. First, hypoplastic amelogenesis imperfecta is characterized by the formation of an enamel matrix with insufficient volume; despite normal mineralization, this results in teeth with a visibly thin and pitted enamel layer [3]. Second, hypocalcified amelogenesis imperfecta is characterized by the normal formation of teeth with enamel that exhibits a low mineral density; this reduced enamel hardness renders the teeth more susceptible to fracture. Third, hypomaturation amelogenesis imperfecta initially results in the formation of enamel in appropriate quantities and with a degree of calcification. However, the enamel crystals themselves are abnormal, leading to the production of easily fractured enamel [10]. Amelogenesis imperfecta is not associated with any systemic diseases. Various classification systems have been proposed over time that emphasize more descriptive and molecularly focused classifications [11].

The investigation and treatment of amelogenesis imperfecta depend on several considerations, including clinical assessment, psychological considerations, and restorative challenges, which are all crucial factors to consider. The patient exhibits a range of oral health issues, including poor oral hygiene due to brushing difficulties, dental caries, discoloration, and shortened clinical crowns [12].

Amelogenesis imperfecta is characterized clinically by a rough enamel surface, and the color is yellow to brown; affected teeth are usually sensitive to temperature as presented and frequently develop large deposits of calculus due to the lack of teeth brushing [13]. On radiographs, enamel mainly appears less opaque than dentin, and a reduction of thickness is usually observed [14]. Most importantly, this case was referred to by the term "partial amelogenesis imperfecta" due to the nature of the problem; the defect was generalized and limited to incisal and middle thirds only. While the cervical third remained intact.

Previous studies have reported similar cases of amelogenesis imperfecta characterized by selective wearing of the incisal and middle thirds of the crown while sparing the cervical third [15-17].

In general, amelogenesis imperfecta patients are recommended to seek dental care at early stages to avert any complications like periodontal disease, severe occlusal wear, altered maxillomandibular relations, caries-related tooth loss, and compromised esthetics [18]. A multidisciplinary treatment approach is greatly needed for more integrated rehabilitation in adults [19]. Treatment objectives are mainly in enhancing the esthetic and avoiding dental sensitivity [15].

In cases of primary teeth, only temporary restorations are allowed when it comes to primary and mixed dentition through conventional/resin-based glass ionomer types of cement, composites, strip crowns, prefabricated metal crowns, and tooth-colored crowns since single porcelain crowns are contraindicated due to the jaw growth and occlusion alterations [20,21]. More precisely, newly introduced high-performance CAD/CAM composites enable the aesthetic replacement of deformed permanent teeth of pediatric patients [21].

A well-formulated treatment plan involving orthodontic, surgical, periodontal, restorative, and prosthetic was planned to improve functional occlusion and enhance good esthetics. Strauch and Hahnel suggested that the type of amelogenesis imperfecta classification is essential for determining treatment options [22]. Several studies proved that indirect porcelain crowns and composite restorations could be a reliable approach even with the presence of a layer of compromised enamel because of the amount of enamel destruction in cases of amelogenesis imperfecta [23,24]. However, other studies revealed that the most common problem recorded in these treatment procedures was debonding [22,25-27]; lower survival rates in direct composite restoration are observed in type II and III amelogenesis imperfecta compared to type I [22]. Indirect porcelain crowns are selected over direct composite restorations; this is due to a higher survival rate compared to direct composite restorations [18].

Comprehensive treatment may involve the patient being referred to a prosthodontist or periodontist. Moreover, orthognathic surgery may be involved in the course of treatment. Esthetic crown lengthening is indicated where there is a gingival overgrowth. Correcting a patient’s occlusion may be achieved by orthodontic treatments before permanent crowns.

## Conclusions

Amelogenesis imperfecta is a genetically based disorder that significantly impacts enamel formation, leading to various clinical challenges, including tooth sensitivity, aesthetic concerns, and increased susceptibility to caries. Prompt diagnosis and intervention are crucial to prevent complications such as periodontal disease and occlusal wear. The clinical findings suggested that this condition may be referred to as "partial amelogenesis imperfecta," as discussed. Continued research into advanced treatment modalities, including computer-aided design and computer-aided manufacturing, may further improve care for these patients in the future.
